# Fucoidan prevent murine autoimmune diabetes via suppression TLR4-signaling pathways, regulation DC/Treg induced immune tolerance and improving gut microecology

**DOI:** 10.1186/s12986-019-0392-1

**Published:** 2019-12-16

**Authors:** Meilan Xue, Hui Liang, Xinqiang Ji, Ying Liu, Yinlin Ge, Lin Hou, Ting Sun

**Affiliations:** 1 Department of Biochemistry and Molecular Biology, Basic Medical College, Qingdao University of Medicine, 38 Dengzhou Road, Qingdao, 266021 People’s Republic of China; 2 The Institute of Human Nutrition, Qingdao University of Medicine, Qingdao, 266021 People’s Republic of China; 3 Department of Gynaecology, the Affiliated Hospital of Qingdao University, Qingdao, 266021 People’s Republic of China

**Keywords:** Type 1 diabetes, Non-obese diabetic mice, Fucoidan, Immune tolerance, Gut microecology

## Abstract

**Background:**

This study was to investigate the effect and its possible mechanism of fucoidan on the development of spontaneous autoimmune diabetes in non-obese diabetic (NOD) mice.

**Methods:**

7-week-old NOD mice were randomly divided into three groups: control group, low-dose (300 mg/kg) and high-dose (600 mg/kg) fucoidan-treatment groups. After 5 weeks of treatment, 10 mice per group were randomly selected to be sacrificed after feces collection. The remaining 12 mice per group were fed until 26 weeks of age to assess the incidence of diabetes.

**Results:**

Treatment with fucoidan increased serum insulin level, delayed the onset and decreased the development of diabetes in NOD mice. Fucoidan reduced the levels of strong Th1 proinflammatory cytokines, but induced Th2-bias ed. cytokine response. And dentridic cells (DCs) in fucoidan treatment group were characterized as low expression of MHC class II and CD86 molecules. TLR4 expressions and the downstream molecules in pancreas were down-regulated in fucoidan-treated groups. There were significant differences in the composition of gut flora between NOD control group and fucoidan group. Lactobacillus and Akkermansia were significantly enriched in fucoidan group.

**Conclusions:**

Fucoidan could prevent the development of autoimmune diabetes in NOD mice via regulating DC/Treg induced immune tolerance, improving gut microecology, down-regulating TLR4 signaling pathway, and maintaining pancreatic internal environment.

## Background

Autoimmune diabetes, also known as Type 1 diabetes mellitus (T1DM), is an autoimmune-mediated disease characterized by selective destruction of insulin-producing pancreatic *β*-cell [1]. The pathogenesis of T1DM relates to genetic factors, autoimmune factors and environmental factors. Based on genetic factors and triggered by environmental factors, it is autoimmune disease characterized by T lymphocytes-mediated progressive damage of islet B cells.

Studies have confirmed that Toll-like receptors (TLRs) are a key family involved in the development of autoimmune inflammation, and inhibition of TLR signaling pathway has great potential in the treatment of autoimmune diseases [2]. In recent years, the role of TLR4 in T1DM has attracted great attention. TLR4 is the main receptor on beta cells and a key molecule that leads to autoimmune damage of beta cells and can serve as an early marker of damage of beta cells [3, 4]. Clinical studies have also shown that TLR4 expression and ligand levels are increased in T1DM patients compared with the control group [5–7]. TLR4 knockout improved the inflammatory state of streptoureasin-induced T1DM model [8].

Regulatory T cells (Tregs) and dendritic cells (DC) are also involved in the pathogenesis of T1DM and play a key role in controlling the progress of the disease. CD4 + CD25+ Tregs could inhibit the differentiation of islet reactive CD8 + T cells into cytotoxic T lymphocytes and prevent the progress of T1DM [9, 10]. Rodent experiments and clinical studies have shown that the gradual loss of Treg inhibition ability is closely related to the development of T1DM [11, 12]. Increasing the differentiation of Treg in NOD mouse model or promoting Treg generation has been proved to be an effective means to combat the occurrence and development of T1DM by protecting beta cells of the pancreas from autoimmune attack. Therapy of type 1 diabetes with CD4(+) CD25 (high) CD127-regulatory T cells prolonged survival of pancreatic islets [13]. Furthermore, the abnormalities in phenotype, maturation and function of DC are related to defective immune regulation of NOD mice and human T1DM [14]. DC also participates in the maintenance of the autoimmune process of T1DM by presenting its own antigen, and mature DC can promote the self-reactive T cell response and reduce the pathogenesis of T1DM [15].

Moreover, as one of environmental factors, gut flora has a direct relationship with the occurrence of type l diabetes by changing intestinal permeability and host immune system [16–18]. Under physiological conditions, gut flora acts as a barrier to intestinal microorganisms, but once the intestinal structure changes, intestinal wall permeability will increase, and then the intestinal immune function also change, which will result in impaired immune tolerance. So microorganisms and anomaly antigens can activate the host immune system through the intestinal barrier, leading to local and systemic inflammatory reaction of target organs. Existing reports have shown that the gut microbiota is associated with the pathogenesis of T1DM in human and non-obese diabetic (NOD) mice [19, 20]. The incidence of T1DM decreased in NOD mice with My88 gene knockout that was given antibiotics to maintain intestinal sterility after intestinal implantation of specific intestinal shade groups, suggesting that gut flora may prevent T1DM. Gut microbial metabolites limit the frequency of autoimmune T cells and protect against type 1 diabetes [21].

Fucoidan, a complex sulfated polysaccharide obtained from brown seaweed, has been widely investigated for its antioxidant, anticancer and anti-inflammatory effects [22, 23]. In vitro and in vivo experiments indicate that fucoidan attenuates hyperglycemia and prevents or impedes the development of diabetic nephropathy related to spontaneous diabetes by attenuating the activation of the NF-κB signaling pathway [24]. Fucoidan can alleviate the inflammatory reaction of P-selection and inflammatory factor, which play a protective role on kidney function of diabetic rats [25]. Recently, fucoidan has been proposed as a potential prebiotic agent for functional food and pharmaceutical development. Shi H, et al. [26] found that dietary fucoidan altered gut flora and repaired the intestinal mucosal injury induced by cyclophosphamide. It is also reported that fucoidan could maintain a more balanced composition of gut flora and reduced the antigen load and the inflammatory response in the host [27]. Our previous studies have examined the effect of fucoidan on intestinal flora and intestinal barrier function in rats with breast cancer. The data showed that dietary supplement of fucoidan could improve the fecal microbiota composition and repair the intestinal barrier function [28]. However, to date and to the best of our knowledge, pathological studies on the effects of fucoidan against autoimmune diabetes in NOD mice have not been carried out.

Fucoidan may regulate intestinal flora and play an effective protective role in T1DM by affecting Treg differentiation and DC phenotype. We attempted to elucidate the molecular mechanism of the protective effect of fucoidan from the perspective of TLR4 signaling pathway. Therefore, NOD mice were used as model animals to conduct in vivo experiments to observe the effect of fucoidan on the pathogenesis of autoimmune diabetes mellitus, and to explore its cellular and molecular mechanisms.

## Methods

### Animals and experimental design

The experiments were carried out according to the National Institutes of Health Guide for Care and Use of Laboratory Animals (Publication No. 85–23, revised 1985). Animal care and the protocols were in accordance with the Animal Experiment Guidelines of Qingdao University of Medicine and ethical approval was obtained from Qingdao University of Medicine.

Male NOD mice at 6 weeks-old were obtained from Beijing Vital River Laboratory Animal Technology Co., Ltd. (Beijing, China). The mice were housed in a controlled environment at a set temperature (22–25 °C) and humidity (50 -60%) and under a 12-h light: dark lighting cycle. All mice were allowed 1 week for acclimatization before experimentation and were allowed free access to standard rodent chow and water throughout the study.

At 7 weeks of age, the animals were randomly divided into three groups: control group, low-dose and high-dose fucoidan-treatment groups. The NOD mice in low-dose and high-dose fucoidan-treatment groups were then given with 300 mg/kg. BW (body weight) or 600 mg/kg.BW fucoidan from *Fucus vesiculosus* (Sigma, St. Louis, MO, USA) respectively by intragastric (i.g.) administration every day. The fucoidan was dissolved in normal saline. The NOD mice in control group were administrated with 0.1 mL normal saline via i.g. per day.

The formula of fucoidan is C_18_H_27_O_21_S_3_--- and its molecular weight is 675.6 KD. As regards the isolation procedure followed by the manufacturer, fucoidans are acid soluble and can be isolated from an algal biomass by simple extraction or by enzymatic digestion. When fucoidan is in solution, it is precipitated with organic solvents using the method described by Black et al. [29]. It is a highly sulphated L-fucose polymer with 95% purity.

After 5 weeks of treatment, 10 mice at 12-weeks of age per group were randomly selected to perform intraperitoneal glucose tolerance test, and then to be sacrificed after feces collection. Blood, spleen and pancreas were collected. One portion of pancreas tissue was kept in formalin solution (10%) for histological examination. The remaining s pancreas tissue was stored immediately at − 80 °C for molecular analysis. One Part of each spleen was used to detect cytokine levels, and the other part of spleen tissue was used to detect CD4 + CD25 + Foxp3+ Treg cells. DC cells were isolated from bone marrow and cultured for 7 days, and then their phenotypes were determined.

The remaining 12 mice per group were fed without fucoidan or saline administration until 26 weeks of age, and the tail vein blood was taken twice a week to assess the incidence of diabetes.

### Intraperitoneal glucose tolerance test (IPGTT)

Mice were given 2 g/kg glucose (200 mg/mL glucose solution) intraperitoneally after fasting for 8 h at night. Blood samples were collected from the caudal vein before (0 h), 0.5 h, 1 h, 2 h and 3 h after the injection, respectively, to determine the blood glucose level. The blood glucose levels were determined using Accu-Chek Performa Blood Glucose Monitor Diabetes Meter and blood glucose test strips (Shanghai Roche Testing Products co. LTD, Shanghai, China).

### Determination of serum insulin, LPS and Th1/Th2 cytokines in spleen

The levels of serum insulin were assessed by ELISA using commercial kits (Cloud-Clone Corp, Houston, USA) according to the manufacturer’s instructions.

The chromogenic end-point Tachypleus amebocyte lysate (CE TAL) assay kit was used to detecte the level of lipopolysaccharide (LPS) in serum and was purchased from Limulus Reagent Rlant Corp (Xiamen, China). The blood was collected in sterile, endotoxin-free tubes. All containers had pyrogen removed by incubating at 180 °C for 24 h. The experiment was conducted in accordance with the manufacturer’s instructions. Finally, the OD was read at 405 nm. The level of LPS was reported in endotoxin units (EU) per milliliter for serum.

ELISA assay was used to detect the levels of spleen cytokines, including IL-1, IL-2, IL-4, IL-6, IL-10, interferon (IFN) -γ and transforming growth factor (TGF) -β. The experiments were performed according to the manufacturer’s protocol (Cloud-Clone Corp, USA).

### CD4 + CD25 + Foxp3+ Tregs analysis

Mouse CD4 + CD25+ Foxp3+ Treg Cells Kit were purchased from eBioscience (San Diego, CA, USA). After the mice were sacrificed, their spleens were quickly removed under aseptic conditions, and part of the spleen tissue was taken to prepare splenic lymphocytes. The spleen tissue was placed in a petri dish containing about 5 mL of serum RPMI-1640 medium (HyClone, Logan, UT, USA), and the spleen was lightly twisted with a sterile needle core to be a single cell suspension. After 100 mesh nylon mesh filtration, the cell suspension under the mesh was collected in the centrifuge tube. After washed with PBS for three times, the cells were adjusted at 106/ml concentration.

The cells were incubated with 0.25 μL FITC-conjugated anti-mouse CD4 and 0.3 μL PE-cy5-conjugated anti-mouse CD25 at room temperature in the dark for 30 min. After washed with flow cytometry staining buffer twice, the cells were permeabilized with fixation/permeabilization solution in the dark for another 30 min and then washed twice with flow cytometry staining buffer. Finally, the cells were stained with 0.5 μL PE-conjugated anti-mouse Foxp3 prior to analysis using a flow cytometer (Becton Dickinson, Franklin Lakes, NJ, USA) to quantify Tregs frequncies. A door was set on CD4, with Foxp3 as the abscissa and CD25 as the ordinate.

### DC isolation and phenotype identification

Bone marrow cells were isolated from femurs and tibiae and cultured for 7 days at a density of 1.5 × 10^6^/mL in RPMI medium containing 10% fetal bovine serum (FBS, Gibco, Carlsbad, California, USA), 20 ng/mL rmGM-CSF (Miltenyi Biotech, Bergisch Gladbach, Germany) and10ng/mL rmIL-4 (Miltenyi Biotech, Bergisch Gladbach, Germany) at 37 °C in a humidified atmosphere with 5% CO_2_. After 3 day of culture, half of the medium was exchanged for fresh medium containing 20 ng/L rmGM-CSF and 10 ng/mL rmIL-4. On day 7, cells were collected and the DC phenotype was determined by flow cytometry. FITC-labeled mouse CD11c antibody, PE-labeled mouse CD86 antibody, and PE-cy5-labeled mouse MHC-II-antibody were added into the 200 μL cell suspension (10^6^/mL), respectively, and incubated in the dark for 30 min. After washed twice with PBS, the cells were resuspended in 400 μL PBS. Phenotype of DC was detected on the flow cytometry. A door was set on CD11c, with CD86 as the X-coordinate and MHC class as the Y-coordinate.

### Western blot analysis

Proteins were extracted from pancreatic tissue by membranal and cytoplasmic Protein Extraction Kit and nuclear and cytoplasmic Protein Extraction Kit (Beyotime Institute of Biotechnology, Jiangsu, China) according to the manufacturer’s instructions. BCA Protein Assay Kit (Beyotime Institute of Biotechnology, Jiangsu, China) was used to determine the protein content. Equal amounts of protein were separated on 5% stacking, 10% SDS-polyacrylamide gels, and subsequently electrotransferred onto PVDF membrane (Solarbio Science & Technology, Beijing, China) at 90 V for 35 min. The membrane was then blocked with 5% non-fat milk, and incubated with specific primary antibodies overnight at 4 °C.

Samples of cell membrane proteins were used to detect the expression of TLR4. Na, K ATP-ase was used as a reference for determination. Samples of cell plasma protein were used to detected the expression levels of myeloid differentiation factor (MyD)88, interleukin (IL)-1β, Toll–IL-1 receptor domain-containing adaptor inducing interferon-β (TRIF), interferon (IFN)-β, LC3 B, p-AMPK, p-mTOR1 inhibition and transcription factor EB (TFEB). β-actin was used as a reference. The nuclear protein samples were used to detect the expression levels of nuclear factor (NF)-κB p65 and interferon regulatory factor (IRF)-3. Histone H3 was used as a reference for nucleoprotein determination. The antibodies for NF-κB p65, IL-1β, LC3 B and insulin were purchased from Cell Signaling Technology in Danvers, MA, USA. The other antibodies were purchased from Proteintech in Rosemont, IL, USA.

After washing with Tris-buffered saline (TBS) for 10 min three times, the membranes were incubated with corresponding secondary antibody (Zhongshan Goldenbridge Biotechnology, Beijing, China, diluted 1/1000) for 1 h. The membranes were washed and detection was carried out with an ECL Western blotting kit (Pierce, Rockford, IL, USA) according to the manufacturer’s instructions.

### Immunofluorescence assay

After dewaxed into water, the pancreatic tissue section was placed in the restoration solution, repaired under high pressure for 5 min, and slowly cooled to room temperature. Then 3% peroxide was added and the sections were incubated at room temperature for 20 min. After blocked in 1% BSA for 1 h, the sections were incubated overnight with primary antibody (insulin, NF-κB p65 and IRF-3) at 1: 60 dilutions. The sections were washed with PBS for 3 times, and incubated with fluorescent second antibody (1: 60) at 37 °C avoid light for 30 min. The DAPI dyeing solution was stained at room temperature for 20 min. After sealed with water-soluble tablet sealing liquid, the sections were observed and photographed by fluorescence microscope.

### Fecal DNA extraction and 16S rDNA gene sequencing

The 16S rDNA gene high-throughput sequencing procedure was performed at the Realbio Genomics Institute (Shanghai, China) by using the Illumina HiSeq platform. The fecal microbiome for 19 fecal samples collected from 9 mice in control group, 10 mice in 600 mg/kg.BW fucoidan treatment group were examined using the Illumina HiSeq 250 platform as described previously [30]. Briefly, the total genomic DNA was extracted from frozen feces using QIAamp DNA Stool Mini Kit (Qiagen, Hilden, Germany) according to the manufacturer’s protocol. The 16S V3-V4 region was amplified using the primers F341 (CCTACGGGRSGCAGCAG) and R806 (GGACTACVVGGGTATCTAATC). The raw data were then subjected to a quality control procedure using UPARSE. USEARCH was used to filter chimeras and the remaining sequences were clustered to generate operational taxonomic units (OTUs) at the 97% similarity level. A representative sequence of each OTU was assigned to a taxonomic level in the RDP database using the RDP classifier. To eliminate the differences caused by variations in the sequencing depth among samples, the least number of sequences obtained were picked randomly for each sample and used for subsequent bioinformatics analysis.

### Statistical analysis

Variance analyses were performed by ANOVA with Tukey’s post hoc test. *t* test was used for comparison the differences between the two groups. Principal components analysis and heat map analysis were conducted with R3.1.0 Differences with *P* values < 0.05 were considered significant.

## Results

### Effects of fucoidan on glucose tolerance, the incidence of diabetes, serum insulin and LPS levels in NOD mice

**Fig. 1:**
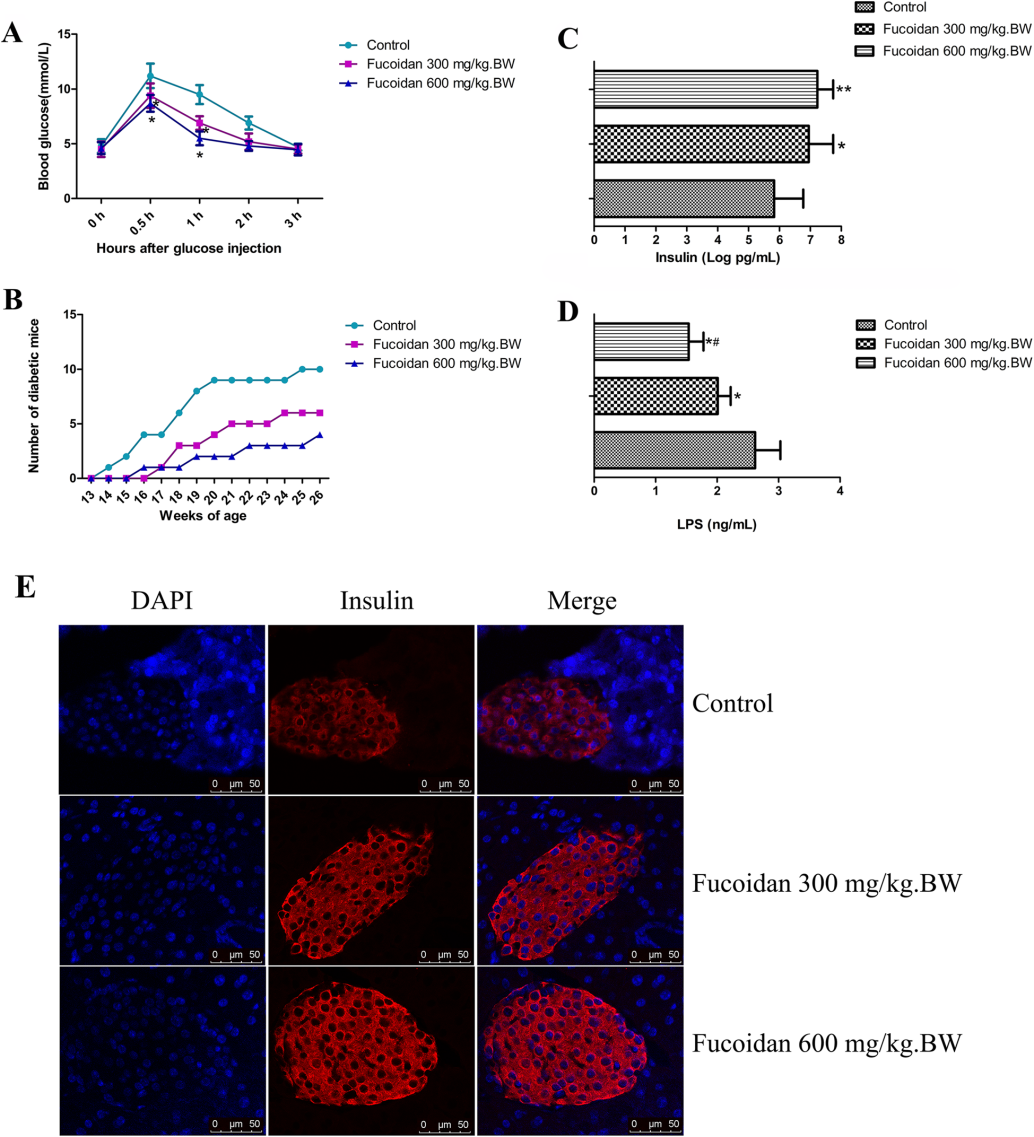
Effect of fucoidan on glucose tolerance, the incidence of diabetes, serum insulin and LPS levels in NOD mice. **a** After 5 weeks of intervention in each group, intraperitoneal glucose tolerance was determined in 12-week-old NOD mice. Compared with the NOD control group, fucoidan treatment (300 mg/kg.BW or 600 mg/kg.BW) significantly lowered blood glucose levels at 30 min and 60 min after glucose load (*P* < 0.05). **b** The blood glucose changes in different week-age mice. The results showed that glucose tolerance was significantly increased. After the intervention, blood glucose was measured twice a week until the animal was 26 weeks old. Among the 12 animals observed in each group, 10 mice in the control group developed diabetes (the incidence rate is 83.3%). Six mice in low-dose fucoidan group developed diabetes (the incidence rate is 50%); Only 4 mice in high-dose fucoidan group developed diabetes (the incidence rate is 33.3%). **c** Serum insulin levels. Compared with NOD control mice, serum insulin levels in 12-week-old NOD mice was increased in high-dose fucoidan groups. **d** Serum LPS levels. After fucoidan intervention, LPS levels decreased. **e** Pancreatic tissue insulin expression. Pancreatic immunofluorescence results showed high insulin expression levels of islet cells in fucoidan treatment groups. *, Compared with the control group, *P* < 0.05; **, Compared with the control group, *P* < 0.01; ^#^, Compared with the 300 mg/kg.BW fucoidan intervention group, *P* < 0.05

The glucose tolerance was determined in mice at 12 weeks of age. As shown in Fig. 1a, compared with the NOD control group, fucoidan treatment (300 mg/kg.BW or 600 mg/kg.BW) significantly lowered blood glucose levels at 30 min and 60 min after glucose load (*P* < 0.05). The results showed that glucose tolerance was significantly increased.

After the intervention, blood glucose was measured twice a week until the animal was 26 weeks old. Among the 12 animals observed in each group, 10 mice in the control group developed diabetes (the incidence rate is 83.3%). Six mice in low-dose fucoidan group developed diabetes (the incidence rate is 50%); Only 4 mice in high-dose fucoidan group developed diabetes (the incidence rate is 33.3%). It suggests that fucoidan could prevent or delay the development of diabetes in NOD mice (Fig. 1b, *P* < 0.05).

Compared with NOD control mice, serum insulin levels in 12-week-old NOD mice was increased in fucoidan groups (Fig. 1c). After fucoidan intervention LPS levels were decreased (Fig. 1d). Pancreatic immunofluorescence results showed that high insulin expression levels of islet cells in fucoidan treatment groups (Fig. 1e).

### Effect of fucoidan on the levels of inflammation in NOD mice

**Fig. 2:**
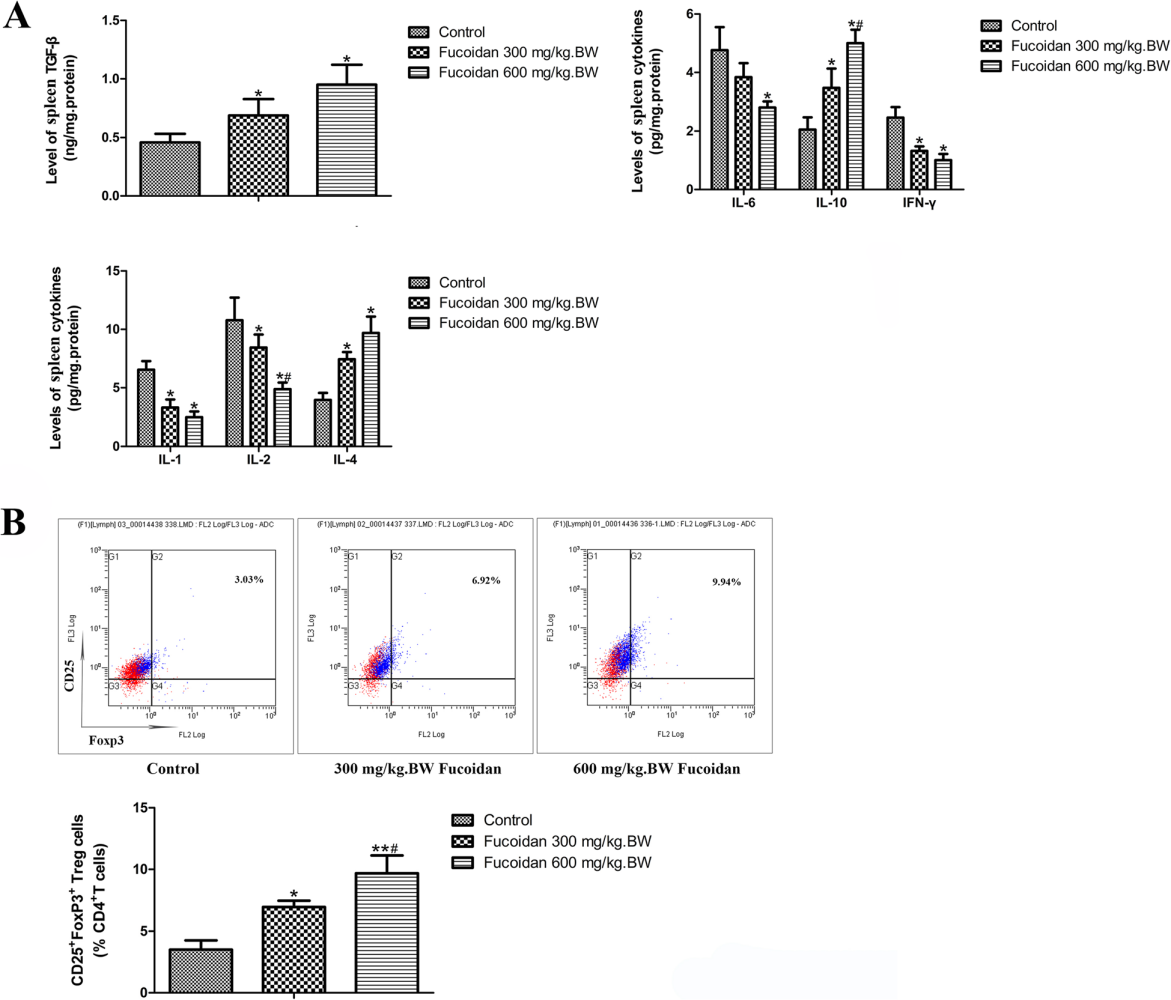
Effects of fucoidan on the levels of cytokines and Tregs in spleen in NOD mice. **a** levels of cytokines in spleen. After intervention with 300 mg/kg.BW and 600 mg/kg.BW fucoidan for 5 weeks, the levels of Th1 type cytokines, IL-1, IL-2, IL-6 and IFN-γ in the spleen of 12-week-old NOD mice were lower than those in the control group. But the levels of Th2 anti-inflammatory cytokines, IL-4, IL-10 and TGF-β were significantly elevated, especially in the high dose fucoidan group. **b** CD4 + CD25 + Foxp3 + Tregs were detected by flow cytometry. Fucoidan intervention could significantly promote the differentiation of CD4 + CD25 + Foxp3 + Treg in spleen lymphocytes. *, Compared with the control group, *P* < 0.05; **, Compared with the control group, *P* < 0.01; ^#^, Compared with the 300 mg/kg.BW fucoidan intervention group, *P* < 0.05

As shown in Fig. 2a, after intervention with 300 mg/kg.BW and 600 mg/kg.BW fucoidan for 5 weeks, the levels of Th1 type cytokines, IL-1, IL-2, IL-6 and IFN-γ in the spleen of 12-week-old NOD mice were lower than those in the control group. But the levels of Th2 anti-inflammatory cytokines, IL-4, IL-10 and TGF-β were significantly elevated, especially in the high dose fucoidan group. It showed that fucoidan could down-regulate Th1 cell-mediated autoimmune response, and induce Th2 cells to produce immunosuppressive cytokines.

**Fig. 3:**
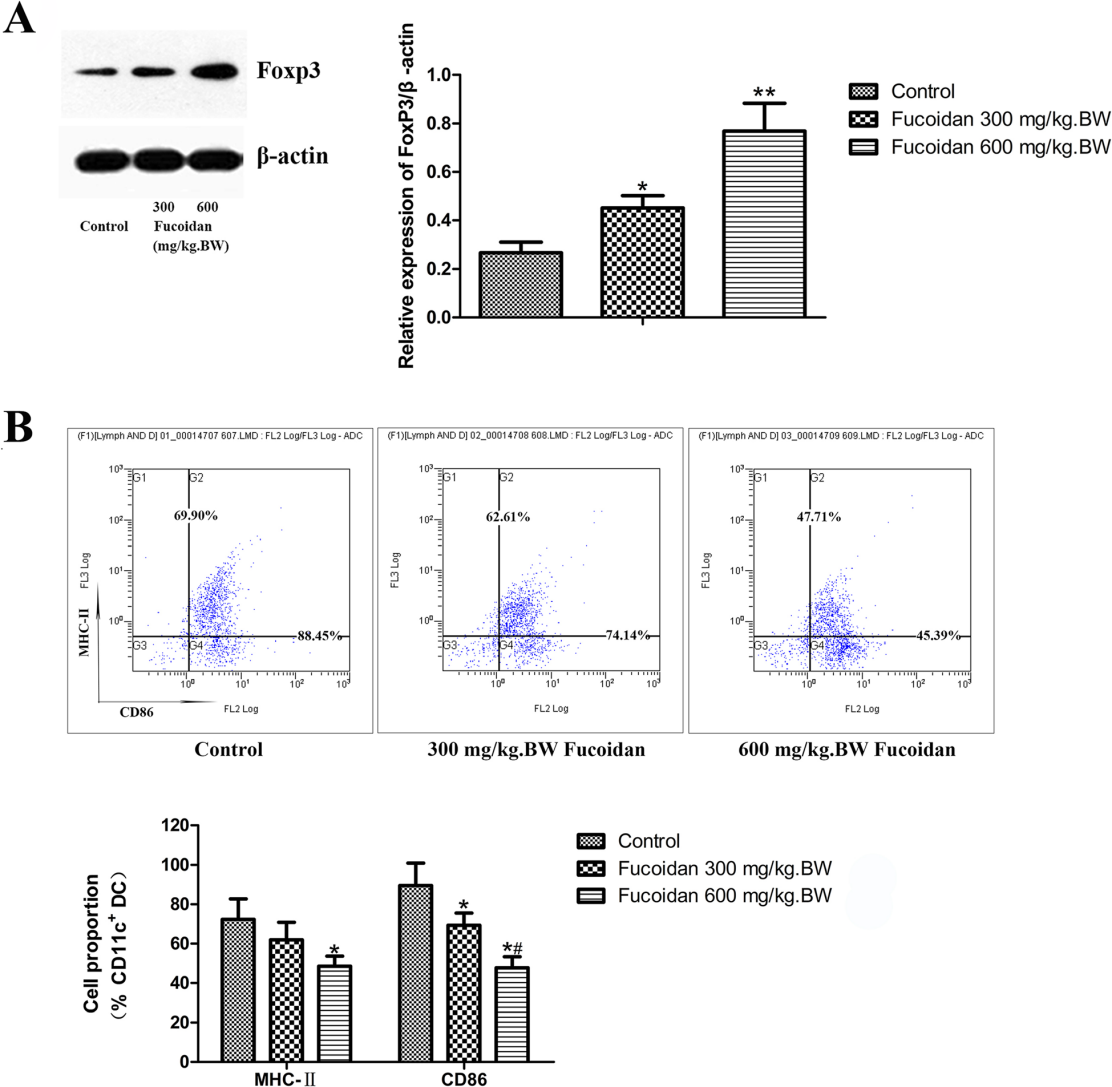
Effects of fucoidan on Foxp3 expression in spleen and DC phenotype in NOD mice. **a** Western Blot analysis of Foxp3 expression in spleen. Fucoidan intervention up-regulated spleen Foxp3 levels. **b** Effect of fucoidan on DC phenotype in NOD mice. Flow cytometry was used to analyze the expression of MHC-II and costimulatory molecule CD86 on DC surface. The expressions of MHC II and CD86 in CD11c + DCs in the 12-week-old NOD mice in the fucoidan intervention group was significantly lower than those in the control group. Especially in the high-dose fucoidan group, the effect was more pronounced. The expressions of MHC II and CD86 in CD11c + DCs in the 12-week-old NOD mice in the fucoidan intervention group was significantly lower than those in the control group. *, Compared with the control group, *P* < 0.05; **, Compared with the control group, *P* < 0.01; ^#^, Compared with the 300 mg/kg.BW fucoidan intervention group, *P* < 0.05

The proportion of CD25+ Foxp3+ Tregs in spleen CD4+ T cells of 12-week-old NOD mice was determined by flow cytometry. As shown in Fig. 2b, CD4 + CD25 + Foxp3+ Tregs in the control group was less differentiated, but the fucoidan intervention could significantly promote the differentiation of CD4 + CD25 + Foxp3 + Treg in spleen lymphocytes (*P* < 0.05). It shown that fucoidan intervention has the effect of inducing immune tolerance. Moreover, compared with the control group, fucoidan intervention up-regulated spleen Foxp3 levels (Fig. 3a, *P* < 0.01). The above results suggest that fucoidan could induce the differentiation of CD4+ CD25+ Foxp3+ T cells in vivo to promote the formation of immune tolerance.

Flow cytometry was also used to analyze the expression of MHC-II and costimulatory molecule CD86 on DC surface. CD11c is a DC landmark marker. The results showed that the expressions of MHC II and CD86 in CD11c + DCs in the 12-week-old NOD mice in the fucoidan intervention group was significantly lower than those in the control group. Especially in the high-dose fucoidan group, the effect was more pronounced (Fig. 3b). It suggested that fucoidan could inhibit the expression of MHC II and CD86 on DC surface, maintain the immature state of DC, and induce immune tolerance in NOD mice.

### Effect of fucoidan on TLR4 pathway in pancreas of NOD mice

To clarify the molecular mechanism by which fucoidan exerts a protective effect on T1DM, we used Western blot to determine the expression of TLR4 protein in pancreatic tissue. The results showed that fucoidan treatment could significantly down-regulate the expression of TLR4 protein.

**Fig. 4:**
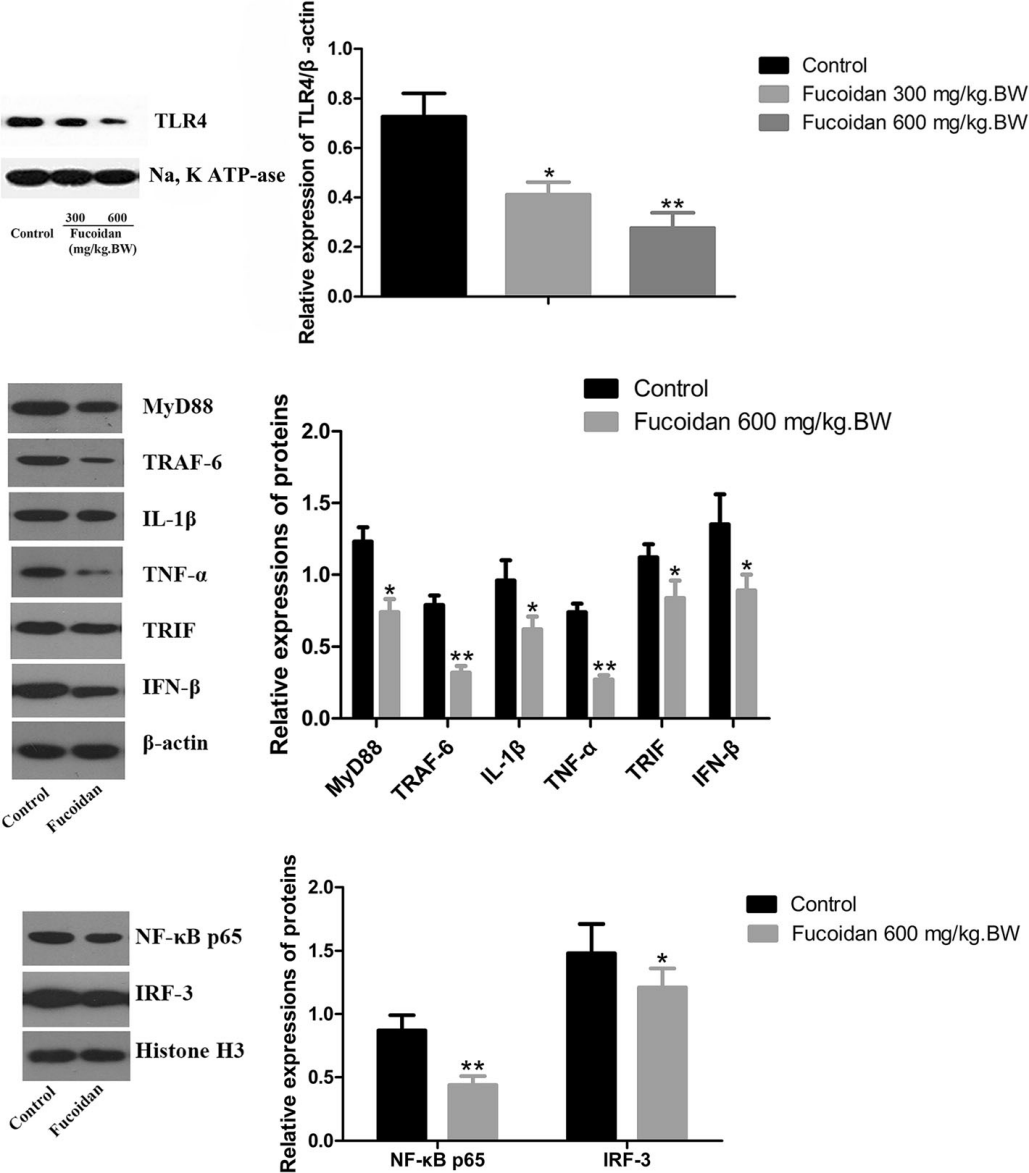
Effect of fucoidan on down-regulation of MyD88-dependent and independent signaling pathways in pancreatic TLR4 in NOD mice. Western results showed that the expressions of MyD88, NF-κB p65 and IL-1β in pancreatic tissue were significantly down-regulated after 600 mg/kg.BW fucoidan intervention. In addition, after 600 mg/kg.BW fucoidan intervention, the expressions of TRIF, IRF-3 and IFN-βin pancreatic tissue were significantly lower than those of the control group. *, Compared with the control group, *P* < 0.05; **, Compared with the control group, *P* < 0.01

**Fig. 5:**
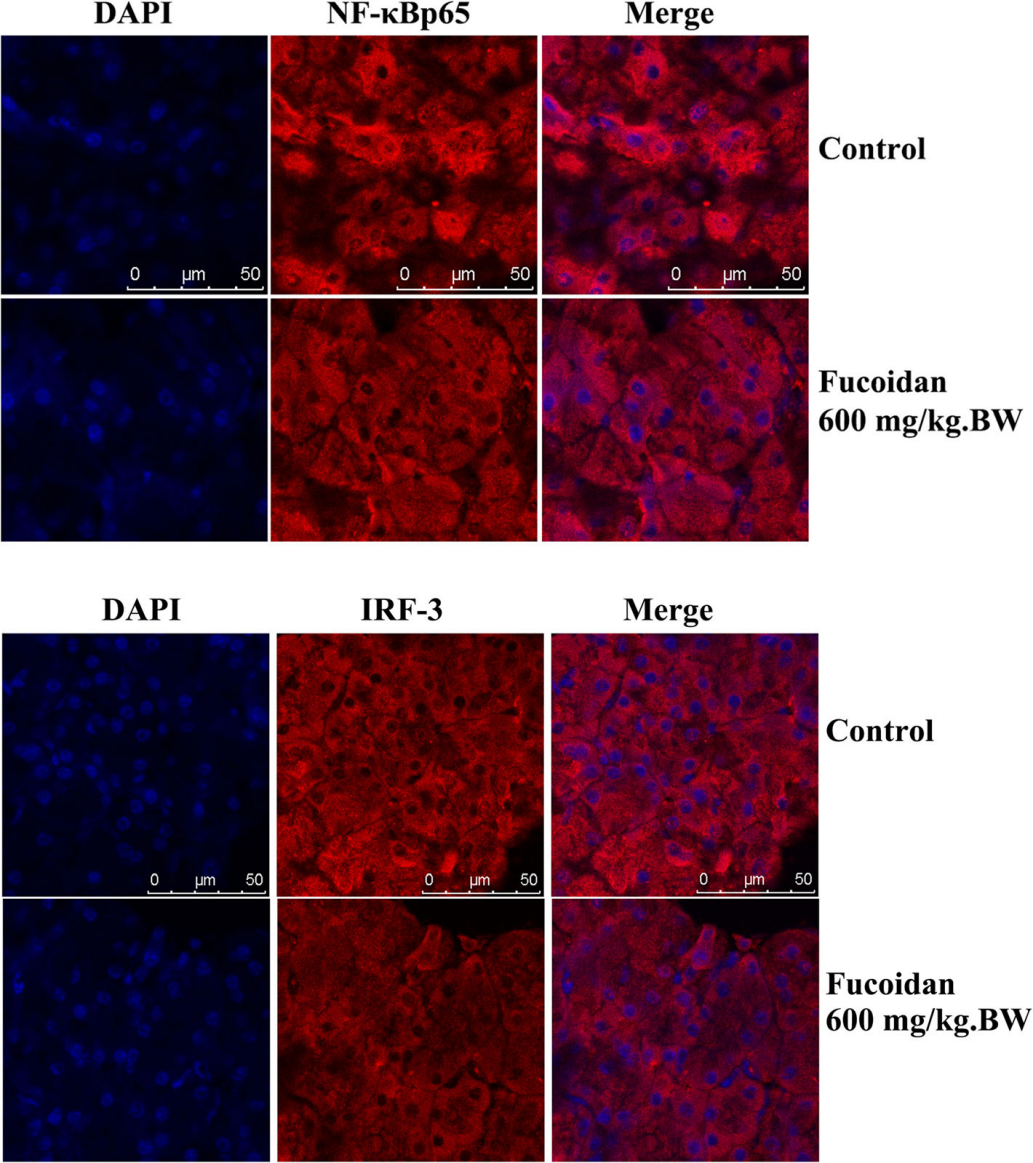
Immunofluorescence assay of NF-κB p65 and IRF-3. Immunofluorescence assay showed reduced nuclear localization of NF-κB p65 and IRF-3 in fucoidan treatment group

To determine whether these two TLR4 downstream signaling pathways--- MyD88 dependent pathway and TRIF dependent pathway were involved in the protective effect of fucoidan on T1DM, we further examined the expression level of TLR4 downstream signaling molecules in pancreatic tissue of NOD mice after fucoidan intervention. Western results showed that the expressions of MyD88, NF-κB p65 and IL-1β in pancreatic tissue were significantly down-regulated after 600 mg/kg.BW fucoidan intervention (Fig. 4). In addition, after 600 mg/kg.BW fucoidan intervention the expressions of TRIF, IRF-3 and IFN-β---the key molecules of TRIF-dependent signaling pathway in pancreatic tissue, were significantly lower than those of the control group (*P* < 0.05; *P* < 0.01). Immunofluorescence assay also showed reduced nuclear localization of NF-κB p65 and IRF-3 in fucoidan treatment group (Fig. 5). The data showed that fucoidan downregulated TLR4-mediated MyD88-dependent signaling pathway and TLR4-mediated TRIF-dependent signaling pathway.

### Effect of fucoidan on intestinal flora of NOD mice

**Fig. 6:**
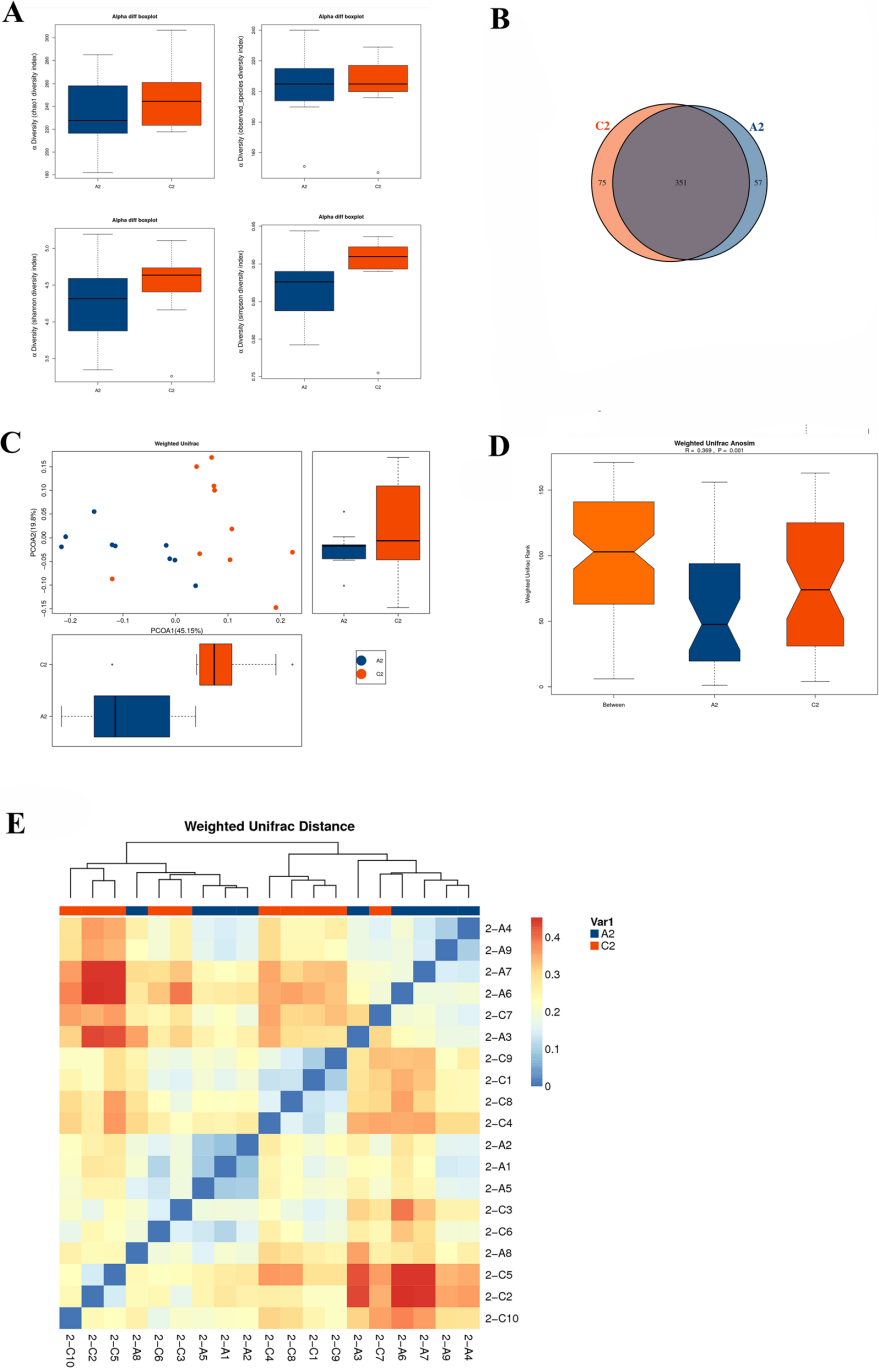
Alpha diversity analysis and Beta diversity analysis in gut flora of NOD mice. **a** Alpha diversity analysis in gut flora of NOD mice. There was no significant difference in the Chao1 index, observed_species index, Shannon index and Simpson index between the NOD control group and the 600 mg / kg. BW fucoidan intervention group. **b** Venn diagram of bacterial OTU; **c** PCA analysis based on OTU abundance; **d** Anosim analysis map; **e** beta diversity heat map. In the Venn, the number of OTUs hold in common in the NOD control group and the 600 mg/kg.BW fucoidan intervention group was 351. The number of unique OTUs is 57 and 75 respectively. According to PCA analysis, there was a difference in microbial composition between the two groups. Based on weighted Unifrac clustering beta diversity analysis (Anosim), the results showed that the composition of the gut flora in the two groups was significantly different (*R* = 0.369, *P* = 0.001). At the OTU level, there was a significant difference in the gut flora structure between the control group and the fucoidan intervention group. A2: NOD control group C2: 600 mg/kg. BW fucoidan intervention group

We used Illumina Miseq high-throughput sequencing technology to sequence the 16S rDNAV3-V4 region of the gut flora of NOD mice. The OTU abundances of each species was shown in Additional file 1. The alpha diversity index of the gut flora in NOD mice was shown in Fig. 6. There was no significant difference in the Chao1 index, observed_species index, Shannon index and Simpson index between the NOD control group and the 600 mg / kg. BW fucoidan intervention group. This indicated that the difference in gut flora diversity between the two groups was not obvious.

In the Venn, the number of OTUs hold in common in the NOD control group and the 600 mg/kg.BW fucoidan intervention group was 351. The number of unique OTUs is 57 and 75 respectively (Fig. 6a). According to PCA analysis, there was a difference in microbial composition between the two groups (Fig. 6b). Based on weighted Unifrac clustering beta diversity analysis (Anosim), the results showed that the composition of the gut flora in the two groups was significantly different (*R* = 0.369, *P* = 0.001). At the OTU level, there was a significant difference in the gut flora structure between the control group and the fucoidan intervention group (Fig. 6c and d).

**Fig. 7:**
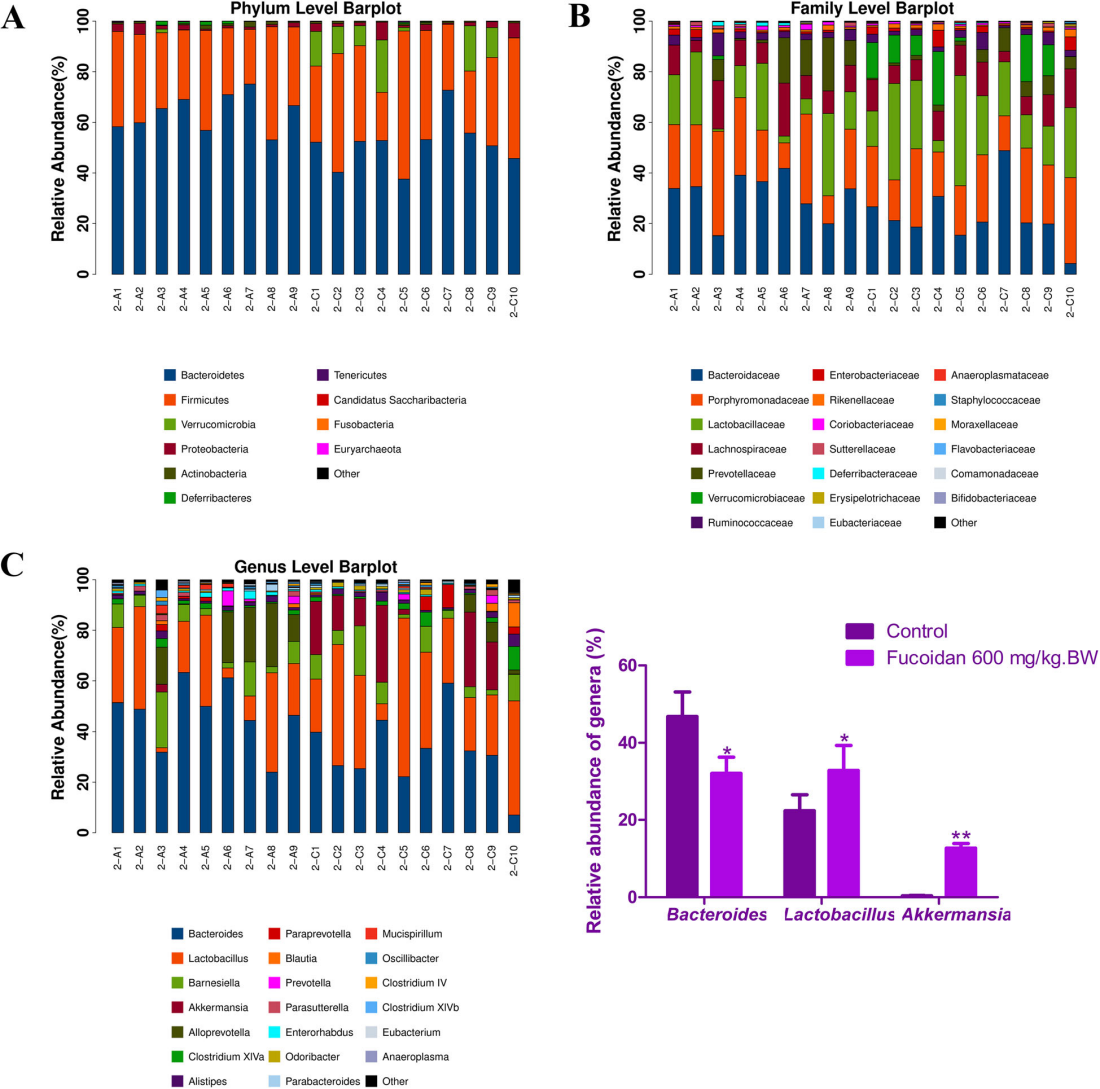
Distribution of gut flora in NOD mice. **a** Analysis of the composition of bacteria at the phylum level. The most dominant phyla in the two groups were Bacteroidetes and Firmicutes. The abundance of Bacteroides in the fucoidan intervention group was 51.37% and significantly lower than that in the NOD control group (63.97%). In addition, the abundance of Verrucomicrobia was increased in fucoidan intervention group (8.41%), but only 0.18% in the control group. **b** Analysis of the composition of bacteria at the family level. The abundances of Bacteroidaceae and Prevotellaceae were 22.6 and 4.00%, respectively, and declined significantly after fucoidan intervention compared with the NOD control group. The abundance of Lactobacillaceae rose to 22.80%, compared with 16.00% in the control group. **c** Analysis of the composition of bacteria at the genus level. Bacteroides was the most dominant genus (46.84%) in the NOD control group, however, the abundance of Bacteroides in the fucoidan intervention group was down-regulated (32.09%), and Lactobacillus became the dominant genus (32.82%). The apparent enrichment of Akkermansia occurred in the fucoidan intervention group, reaching to 12.69%, and the control group was only 0.37%. Other than that, the fucoidan intervention also increased the abundance of Clostridium XlVa and Anaerofustis, while the abundance of Alloprevotella, Enterorhabdus, and Mucispirillum was reduced. A2: NOD control group; C2; 600 mg / kg. BW fucoidan intervention group

Cluster analysis showed that the composition of the gut flora in control group and the 600 mg/kg.BW fucoidan intervention group was significantly different. The most dominant phyla in the two groups were Bacteroidetes and Firmicutes. The abundance of Bacteroides in the fucoidan intervention group was 51.37% and significantly lower than that in the NOD control group (63.97%). In addition, the abundance of Verrucomicrobia was increased in fucoidan intervention group (8.41%), but only 0.18% in the control group (Fig. 7a). At the family level, compared with the NOD control group, the abundances of Bacteroidaceae and Prevotellaceae were 22.6 and 4.00%, respectively, and declined significantly after fucoidan intervention. But the abundance of Lactobacillaceae rose to 22.80%, compared with 16.00% in the control group (Fig. 7b).

The results of the genus analysis indicated that the genus distribution of the gut flora of the two groups changed significantly. Bacteroides was the most dominant genus (46.84%) in the NOD control group, however, the abundance of Bacteroides in the fucoidan intervention group was down-regulated (32.09%), and Lactobacillus became the dominant genus (32.82%). It was worth mentioning that the apparent enrichment of Akkermansia occurred in the fucoidan intervention group, reaching to 12.69%, and the control group was only 0.37%. Other than that, the fucoidan intervention also increased the abundance of Clostridium XlVa and Anaerofustis, while the abundance of Alloprevotella, Enterorhabdus, and Mucispirillum was reduced (Fig. 7c).

### Spearman correlation analysis between genus species and serological indicators

**Fig. 8:**
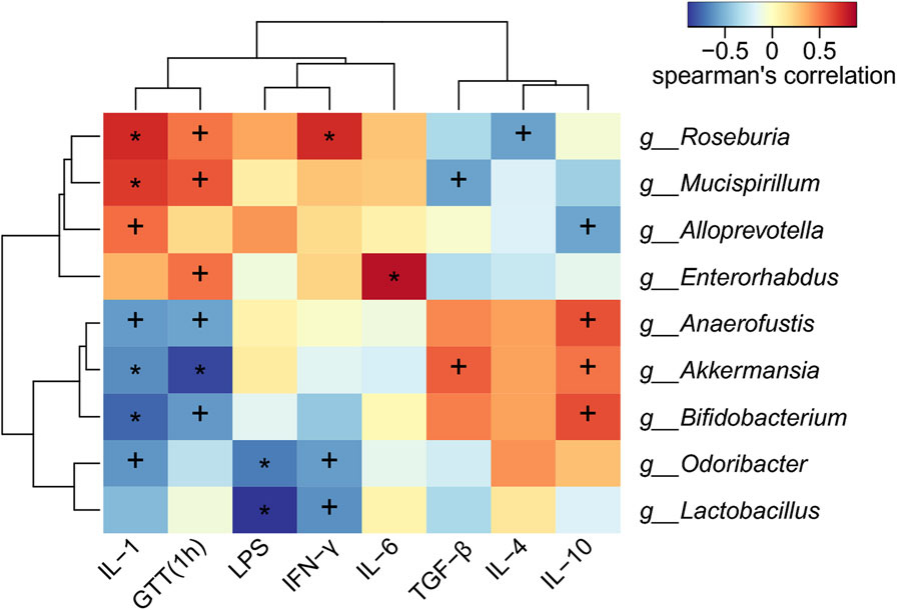
Spearman correlation analysis between genus species and serological indicators. The abundance of Lactobacillus was negatively correlated with the LPS and IFN-γ. The abundances of Akkermansia and Anaerofustis were negatively correlated with blood glucose of GTT (1 h) and IL-1, and positively correlated with the level of IL-10. In addition, the blood glucose of GTT (1 h) was positively correlated with the abundances of Enterorhabdus, and Mucispirillum. The abundances of Alloprevotella was positively correlated with the level of IL-IL-1, and negatively correlated with IL-10. The results indicated that fucoidan may reduce the level of inflammation and improve the glucose tolerance by regulating gut flora. X-axis, serological indicators; Y-axis, genus species. The depth of color visually shows the correlation between genus species and serological indicators. ^+^, *P* < 0.05; *, *P* < 0.01

Spearman correlation analysis between genus species and serological indicators was shown in Fig. 8. The abundance of Lactobacillus was negatively correlated with the LPS and IFN-γ. The abundances of Akkermansia and Anaerofustis were negatively correlated with blood glucose of GTT (1 h) and IL-1, and positively correlated with the level of IL-10. In addition, the blood glucose of GTT (1 h) was positively correlated with the abundances of Enterorhabdus, and Mucispirillum. The abundances of Alloprevotella was positively correlated with the level of IL-IL-1, and negatively correlated with IL-10. The results indicated that fucoidan may reduce the level of inflammation and improve the glucose tolerance by regulating gut flora.

## Discussion

In this study, we found that treatment with fucoidan for 5 weeks significantly increased insulin levels, improved the glucose tolerance, delayed the onset and decreased the development of diabetes by 26 weeks of age in NOD mice. Fucoidan reduced the levels of strong Th1 proinflammatory cytokines, but induced Th2-biased cytokine response, the generation of CD4 + CD25+ Foxp3+ Tregs in spleen and Foxp3 expression in pancreas. And fucoidan-treated DCs were characterized as low expression of MHC class II and CD86 molecules.

T1DM is an autoimmune disease in which the absence of autoimmune tolerance causes specific damage to beta cells of the pancreas. Th1-mediated autoimmune disease is involved in T1DM [31, 32]. The relationship between T1DM and high levels of inflammatory cytokines such a TNF-훼, interferon- (IFN-) 훾 and IL-1β has been widely recognized. Increasing Th2-type cytokines (such as IL-10, TGF-β), and at the same time, reducing the production of Th1 type cytokines (such as IL-2, IFN-γ) are ideal means to prevent and control T1DM. CD4 + CD25 + Foxp3+ Treg cells control immune responses and maintain immunological tolerance [33]. These cells regulate cytokine production (IL-10 and TGF-β), modification of dendritic cell (DC) function (downregulation of co-receptors CD80/86), and cytokine deprivation (through sequestering of IL-2 by CD25) [34]. Foxp3 is extremely important for the differentiation and function of Treg. Loss of Foxp3 expression produces inflammatory cells and is involved in the of T1DM [35]. Immune responses via several mechanisms including suppressive CD4 + CD25 + Foxp3+ Treg has an immunomodulatory effect, can fight the development of autoimmune diabetes. Our data suggested that fucoidan could induce the differentiation of CD4 + CD25 + Foxp3 pathogenesis + T cells in vivo, inhibit the expression of MHC II and CD86 on DC surface, down-regulate Th1 cell-mediated autoimmune response, and induce Th2 cells to produce immunosuppressive cytokines, that resulted in immune tolerance in NOD mice.

In order to clarify the mechanism of immune tolerance mediated by fucoidan, we investigated the effect of fucoidan on TLR4 and its related pathway molecules. There are two signaling pathways downstream of the TLR4 signaling pathway, MyD88 dependency and TRIF dependency (MyD88 independency) [36]. The TIR domain of TLR4 interacts with the adaptor protein MyD88. Once stimulated, TLR4 can bind to MyD88, activate TRAF-6, and cause the transcription factor NF-κB to activate into the nucleus, that leads to natural immune and inflammatory reactions including the production of the proinflammatory cytokines IL-1β and TNF-α. TRIF is another molecule containing a TIR domain, that plays an important role in the MyD88-independent signaling pathway downstream of TLR4 [37]. TRIF is essential in the process of stimulating IFN-β production via IRF-3 (An essential DNA-binding transcriptional activator protein). Our results showed that fucoidan could down-regulated TLR4-mediated MyD88 dependent and TRIF-dependent signaling pathway.

Moreover, gut flora plays a key role in regulating host metabolism, immunity and inflammation [38]. Intestinal flora imbalance is associated with various diseases, including obesity, diabetes, atherosclerosis, high blood pressure and so on. Gut microbial metabolites limit the frequency of autoimmune T cells and protect against type 1 diabetes [21]. In mice of the NOD strain, the researchers found that key features of disease correlated inversely with blood and fecal concentrations of the microbial metabolites, acetate and butyrate. Miani M, et al. [39] revealed that gut microbiota conditioned innate lymphoid cells (ILCs) induce the expression of mouse β-defensin 14 (mBD14) by pancreatic endocrine cells, preventing autoimmune diabetes in the NOD mice.

The data in this research showed that there were significant differences in the composition of gut flora between NOD control group and fucoidan treatment group. The abundance of Bacteroides phylum in the fucoidan intervention group was decreased, while the abundance of Verrucomicrobia phylum was increased. At the family level, the abundances of Bacteroidaceae and Prevotellaceae were declined significantly after fucoidan intervention. But the abundance of Lactobacillaceae rose to 22.80%. It has reported that Bacteroides in the gut flora is a major producer of branched-chain amino acids, increased serum branched-chain amino acids lead to increased insulin resistance [40, 41]. Bacteroides was the most dominant genus in the NOD control group, however, in the fucoidan intervention group, the abundance of Bacteroides was down-regulated, and Lactobacillus became the dominant genus. It was worth mentioning that the apparent enrichment of Akkermansia occurred in the fucoidan intervention group. In addition, fucoidan also increased the abundances of Clostridium XlVa and Anaerofustis, and decreased the abundances of Alloprevotella, Enterorhabdus and Mucispirillum. Moreover, the abundance of Lactobacillus was negatively correlated with the LPS and IFN-γ. The abundances of Akkermansia and Anaerofustis were negatively correlated with blood glucose of GTT (1 h) and IL-1, and positively correlated with the level of IL-10. Studies have shown that Lactobacillus can induce the secretion of IL-10, and prevention of T1DM by regulatory T cells [42]. Lactobacillus casei can alter the shape of dendritic cells, making DC more sensitive to IL-10, producing immune tolerance and delaying the development of T1DM [43]. Akkermansia is a significantly reduced flora of diabetic patients and pre-diabetes, feeding live AKK bacteria to high-fat diet mice can reverse metabolic disorders such as insulin resistance [44–46]. Clostridium species have been associated with the number and function of Treg cells in the colon of mice [47]. In this study, after fucoidan intervention Bacteroides was downregulated in NOD mice, but Lactobacillus and Akkermansia were obviously enriched. It indicated that fucoidan may reduce the level of inflammation, improve the glucose tolerance and delay the occurrence of T1DM by regulating gut flora.

In addition, Bacterial species that are protective against diabetes might display qualities through innate signaling molecules, such as LPS [48]. TLR4 is a receptor for LPS, and the pro-inflammatory activity of TLR4 is linked with pathological responses to endogenous ligands in autoimmune disorders [49]. After fucoidan intervention, LPS levels were decreased. It may be one of the mechanisms of fucoidan for down-regulating TLR4 pathway that fucoidan reduced the production of LPS by affecting the gut flora.

## Conclusions

This study suggested that fucoidan could prevent the development of autoimmune diabetes in NOD mice via regulating DC/Treg induced immune tolerance, improving gut microecology, down-regulating TLR4 signaling pathway, and maintaining pancreatic internal environment by enhancing autophagy and inhibiting apoptosis of pancreatic cells.

## Supplementary information

**Additional file 1.** Raw sequence reads of each fecal flora species. The categories and abundance of the bacteria that were detected in the samples were listed in the file.

## Data Availability

All data generated or analysed during this study are included in this published article [and its Additional files].

## Abbreviations

CE TAL: Chromogenic end-point Tachypleus amebocyte lysate
DC: Dendritic cells
EU: Endotoxin units
IFN: Interferon
IPGTT: Intraperitoneal glucose tolerance test
IRF: Interferon regulatory factor
LPS: Lipopolysaccharide
NOD: Non-obese diabetic
T1DM: Type 1 diabetes mellitus
TBS: Tris-buffered saline
TFEB: Transcription factor EB
TGF: transforming growth factor
TLRs: Toll-like receptors
Tregs: Regulatory T cells
